# A predictive model of waterpipe smoking cessation among women in southern Iran: application of the theory of planned behavior

**DOI:** 10.1186/s12889-023-16053-4

**Published:** 2023-06-15

**Authors:** Sara Dadipoor, Gholamreza Heydari, Niveen ME Abu-Rmeileh, Shokrollah Mohseni, Hadi Eshaghi Sani Kakhaki, Teamur Aghamolaei, Nahid Shahabi

**Affiliations:** 1 Tobacco and Health Research Center, Hormozgan University of Medical Sciences, Bandar Abbas, Iran; 2 Tobacco Prevention and Control Research Center, National Research Institute of Tuberculosis and Lung Diseases, Shahid Beheshti University of Medical Sciences, Tehran, Iran; 3 Institute of Community and Public Health, Birzeit University, Birzeit, Palestine; 4 Social Determinants in Health Promotion Research Center, Hormozgan Health Institute, Hormozgan University of Medical Sciences, Bandar Abbas, Iran; 5 Student Research Committee, Hormozgan University of Medical Sciences, Bandar Abbas, Iran

**Keywords:** Water-pipe, Smoking cessation, Theory, Predictor, Iran

## Abstract

**Background:**

Today, waterpipe (WT) smoking is a rising issue worldwide, and has taken a significant and growing share of tobacco consumption in the world. Present study aimed to explore the predictors of WT cessation in the light of the theory of planned behavior (TPB).

**Methods:**

This cross-sectional analytical study was conducted in 2021–2022 using a multi-stratified cluster sampling on 1,764 women in Bandar Abbas, southern Iran. Data were collected through a reliable and valid questionnaire. The three-part questionnaire includes demographic information, behavioral information of WT smoking, and the constructs of the TPB along with an additional habit construct. Multivariate logistic regression analysis was run to model the predictor constructs of WT smoking. The data were analyzed statistically in STATA14.2.

**Results:**

With an increase in one attitude score, the odds of cessation increased by 31% (*p* < 0.001). Also, with an increase of one score in knowledge, the odds of cessation are increased by 0.05% (0.008). With an increase of one score for intention, the odds of cessation are 26% (0.000). in social norms, the odds of cessation are 0.02% (0.001). With an increase of one score in perceived control, the odds of cessation increased by 16% (0.000) and inhabit score, the odds of cessation decrease by 37% (0.000). In the model where the habit construct was present, the accuracy, sensitivity, and pseudo R2 indices were 95.69%, 77.31%, and 65%, respectively and after removing the habit construct, the so-called indices changed to 90.7%, 50.38% and 0.44%, respectively.

**Conclusions:**

The present research confirmed the effectiveness of the TPB model in predicting waterpipe cessation behavior. The knowledge obtained from this research can help develop a systematic and effective intervention to facilitate waterpipe cessation. Focusing on the habit variable can play a critical role in waterpipe cessation in women.

## Background

Cigarette smoking is the dominant form of tobacco consumption in many countries. Yet, waterpipe smoking (WTS) is also common and accounts for a significant and growing share of tobacco use globally. The other common names for waterpipe are hookah, shisha, narghile, arghile. Goza, oriented pipe, Hubble bubble, Mada’s and glaze base [1].

WTS has been raised as an important global issue, especially in Eastern Mediterranean countries such as Arab countries, Turkey and Iran [2]. Eastern Mediterranean regions have the highest prevalence of WTS, which has been on the rise in the last two decades [3–5]. In Iran, the prevalence of WTS has increased significantly, too [6, 7]. A population with a high rate of WTS is women [8–10]. The global prevalence of WTC among women has been reported to be 41% [11]. The rate of WTC is 8.7% among Eastern Mediterranean women [12], 4% among Lebanese women [13], 4% among Pakistani women [11] and up to 16.8% among Iranian women.

Within Iran, in Hormozgan Province, in the south of Iran, the rate of WTC was reported to be 10.3%, which is 9 to 10 times as high as other provinces [14]. Among women in the south of Iran, this rate has been reported to range between 10.3% and 16.8%. In another study in Hormozgan Province, 28.4% of men and 45.2% of women smoked WT [15].

Waterpipe smoking is associated with consequences such as lung disease [16], adverse Covid-19 outcomes [17], myocardial infarction [18], stroke [19]. At the same time, the side effects of WTC are more in women than in men [20]. WTC in women is associated with an increased risk of early menopause, decreased bone density, infertility, ectopic pregnancy, increased disease and infant mortality, intrauterine growth restriction, and an increase in some chromosomal incompatibilities and genital warts [21–23].

The high prevalence of WTS in women, on the one hand, and its side effects, on the other hand, make it more essential than ever to stop this unhealthy habit. It is believed that learning about the predictors of WT cessation can be vital step in helping women to stop WTS. Systematic and effective interventions can be developed based on these variables.

Psychosocial problems are a leading cause of smoking, so theory-based interventions with a focus on self-efficacy, motivation, beliefs and knowledge of consumers to cease smoking have a significant effect [24]. Intention to cease WTS among smokers is a strong predictor of actual attempts to cease WTS [25]. A study reported that perceived behavioral control over smoking cessation is a central mechanism underlying the relationship between nicotine dependence and intention to cease WTS [26]. Social media is another effective factor in WTS cessation [27]. Currently, there is no conceptual model that takes into account all the above-mentioned variables in cessation.

The theory of planned behavior (TPB) has been used successfully in predicting and making sense of several health-related behaviors including tobacco smoking [28, 29]. Models of behavior, such as the TPB provide a conceptual framework that allows program designers and policy makers to detect the fundamental features that determine behavior and, thus, develop effective interventions [30].

### Theoretical framework

The theory of planned behavior (TPB) is a theory commonly used in developing or assessing smoking cessation programs. In this theory, the main determinants of a behavioral intention are the attitude, subjective norms, and perceived control over a behavior. Attitude refers to beliefs about the probable outcomes of the specific behavior and the assessment of these outcomes (or the behavioral beliefs). The other determinant, subjective norms, is based on beliefs about the others’ normative expectations and motivation to meet these expectations (or normative beliefs), and perceived control over behavior is manifested as beliefs about the underlying effect of the variables which can facilitate or prevent the adoption of that certain behavior and the perceived impact of these variables (or control beliefs) [31].

Upon developing the TPB, Ajzen declared that the theory is basically open to the addition of other determinants only if they add to the variance explained in behavioral intentions [32]. This research used the TPB with an additional element, known as habit as an additional predictor of behavioral intentions.

**Fig. 1:**
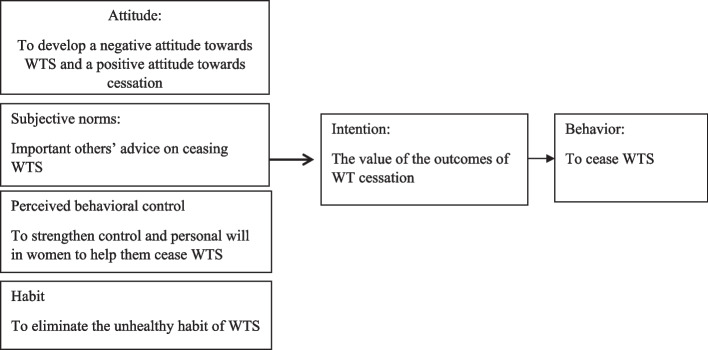
Theoretical model (theory of planned behavior) used for WT cessation

In the existing literature, the habit (physical-psychological dependence) of smoking was reported as one of the important barriers to smoking cessation [33, 34]. Obtaining this information is the first step in planning and implementing anti-smoking programs. The conceptual theory is shown in Fig. 1.

### Review of the related literature

In previous studies, the TPB has been successfully used in predicting smoking cessation. Yu-Fang Tseng et al. used TPB to predict smoking cessation. They found the TPB a useful framework for smoking cessation intention. In the same study, the TPB constructs with an additional construct of perceived susceptibility predicted 40% of the variance of smoking cessation [29]. Paul Norman et al. found that the intention to cease smoking is predicted by perceived behavioral control and perceived susceptibility [35]. Other studies confirmed the effectiveness of educational interventions based on the TPB in preventing and reducing smoking among women [36, 37]. A systematic study reported that the TPB constructs were effective in 42%-50% of smoking behaviors [38]. According to the reviews, few studies examined the predictors of WTS [39, 40], or explored the predictors of WT cessation [41, 42].

Of note is that in most previous studies, the focus was on the intention to cease smoking or on the continued intention of waterpipe smoking. The actual behavior of cessation was not considered. As suggested in the existing literature, it is better to consider the actual behavior of cessation (one limitation of the existing literature) [26, 43, 44]. In the present study, the limitations of previous studies were compensated for and the real behavior of ex-WT-smokers was predicted based on the TPB constructs.

Considering the above-mentioned issues and the effect of wide geographical diversity and gender on the pattern of waterpipe smoking in Iran [45], the present research aimed to explore the predictors of WT cessation in light of the theory of planned behavior (TPB).

### Research hypotheses

1. All TPB constructs predict WT cessation behavior in women.
2. Perceived behavioral control and habit are the strongest predictors of smoking cessation in women.
3. The habit construct increases the predictive power of the TPB model.

## Methods

### Design of study and participants

The present Analytica cross-sectional study was conducted in 2021–2022 in Bandar Abbas, a metropolis in Hormozgan Province in the south of Iran. This city is located in 27.19 latitude and 56.28 longitude and is situated at the height of 9 m above the sea level. Bandar Abbas has a population of 352,173 people, making it the biggest city in Hormozgan Province.

Global facts and figures [8, 10] as well as those in Hormozgan Province show an increase in the rate of WTS in women compared to men [15, 46]. women in Hormozgan Province rank third in the prevalence of WTS in Iran [14]. Therefore, the present study chose to investigate this population.

As the present study aimed to explore the predictors of WT cessation, some women were WT smokers and some were women who had successfully managed to cease WTS.

### Inclusion and exclusion criteria

The inclusion criteria were the women's history of waterpipe smoking within the past year, mastery of the local dialect, being native to Bandar Abbas city and providing an informed and voluntary consent to take part in the research. The exclusion criterion was the cessation for less than 6 months in the cessation group.

Addiction to any type of substance except for WT tobacco. This research was carried out in Hormozgan University of Medical Sciences and the Tobacco Prevention and Control Research Center of Shahid Beheshti University of Medical Sciences, respectively, with an ethical code of IR.HUMS.REC.1401.188.

### Sample size determination and sampling technique

The main objectives of the present cross-sectional study were to determine the prevalence of successful WT cessation and to explain the predictive power of the TPB constructs for the expected outcome (successful cessation or no cessation) using a multivariate logistic regression. To estimate the required sample size, firstly, a pilot study was conducted by the researchers with a sample size of 64. In this pilot test, the frequency of successful cessation was estimated at 10 (15.6% of the total sample). With an accuracy value of $d=2$ and the following formula, the sample size was estimated at 1,264.

$$n=\frac{{z}^{2}pq}{{d}^{2}}=\frac{{\left(1.96\right)}^{2}\left(15.6\right)\left(84.4\right)}{{2}^{2}}=1264$$

The sampling method in this study was clustered. A design effect of 1.5 was considered and the sample size obtained from the above formula was multiplied by the design effect and the final sample size was estimated at 1,896.

In the present study, the women were selected and included through a multi-stratified cluster sampling method. To this aim, in the first step, among the 20 comprehensive health service centers of Bandar Abbas city, 12 were selected as the 12 clusters. In the second step, three alleys were randomly selected as the second clusters. Then in each alley, the first house was chosen as the head of the cluster. From the right side of each head of the cluster, the houses were visited until 53 women who met the inclusion criteria were contacted.

**Fig. 2:**
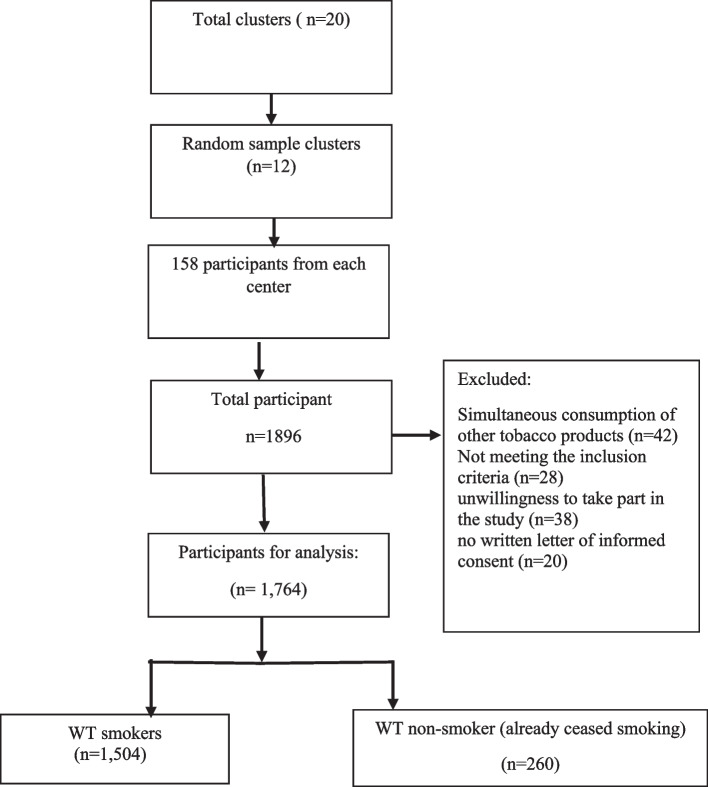
Flow chart of sampling process

The sampling was convenience in type. From each household, all eligible members were included in the study (Fig. 2).

### Survey development, validation and reliability

The data collection instrument was a researcher-made three-part structured questionnaire. The development of this instrument was based on an extensive review of the related literature. All the information was completed as self-reports. The three parts are described below.

Demographic information containing the women’s educational level, occupation, and socioeconomic status (SES)
(WTS) behavioral information containing the age of beginning to smoke WTs, history of WTS, type of tobacco used, WTS by other family members, weekly consumption rate
TPB constructs and the range of scoring, ICC, Cronbach’s alpha coefficient and sample items as indicated in Table 1.

**Table 1:** Item response and reliability of the TPB scale

Construct	No. of Items (Format)	Scoring (Range)	ICC	Cronbach’s alpha coefficient	Sample item
1) Knowledge	10 items/ 3 multiple choice	1. true 2. false 3. don't know	0.804	0.79	WT smoke is purified by the water tank and is not harmful
2) Attitudes	15 items/5 point Likert Scale (strongly disagree- strongly agree)	SD = 1, D = 2, NI = 3, A = 4, SA = 5	0.879	0.84	WTS is harmful to health
3) Perceived social norms(SN)	20 items/5 point Likert Scale (strongly disagree- strongly agree)	SD = 1, D = 2, NI = 3, A = 4, SA = 5	0.785	0.73	My family expects me to replace WT with healthier behaviors
4)Perceived behavior control	9 items/5 point Likert Scale (strongly disagree- strongly agree)	SD = 1, D = 2, NI = 3, A = 4, SA = 5	0.845	0.78	How confident are you that you can break up with WT smoking friends?
5) Habit	7 items //5 point Likert Scale	SD = 1, D = 2, NI = 3, A = 4, SA = 5	0.908	0.86	I can't cease WTS because of consuming it for years
6. Intention	1 Item	1. yes 2. No	0.836		Do you intend to cease WTS now? If you had already quit waterpipe smoking, do you intend to continue the cessation?
7. Behavior	1 Item	1. yes 2. No	0.973		Cessation/no cessation

### Data quality assurance

To test the content validity of the measurement instrument, the questionnaire was provided to 5 experts in the field of health education, 5 physicians trained in smoking cessation and 3 clinical psychologists. Their comments were used to modify the questionnaire. To measure the reliability of the instrument, the test–retest method was used. Thus, the questionnaire was submitted to 30 women in the same conditions as the main participants of study in two weeks and on two occasions. After that, each question in the first test was compared with the second test. If the correlation coefficient between the first and second tests in each section was higher than 0.7, the reliability was confirmed. After that, to calculate the agreement between the test and retest, the ICC index was calculated. Thus, to calculate the agreement between the average test scores and the average retest scores, the ICC index value was estimated at 0.833 and the questionnaire was found to be reliable.

### Data collection

The data were collected daily in the morning and afternoon using a researcher-made questionnaire. The first author, who was trained, local to the area and familiar with the data collection method, completed the questionnaires by visiting the houses in person. Completing each questionnaire took about 15 min. Women who were literate completed the questionnaire at home at their convenience and then returned it to the researcher. For those women who were not literate, the questionnaire content was read out loud and completed by the researcher trying to minimize bias.

### Ethical considerations

For the data collection, the researcher visited the comprehensive health service centers with an official letter of permission from the research assistant of university and then visited the data collection sites. First, the researcher fully introduced herself and explained the objectives of study to the women participants of the target group in simple words. Then, a written letter of consent with all details of study was signed by the women and for those who were minors and illiterates, informed consent has been obtained from a parent and/or legal guardian. They were assured of the confidentiality of the information they provided. This study was approved by the ethics committee of Hormozgan University of Medical Sciences and the Tobacco Prevention and Control Research Center of Shahid Beheshti University of Medical Sciences.

### Data management

In the present study, interval variables (age, knowledge, perceived behavioral control, habit, intention, attitude, social norms) were measured and reported using descriptive statistics (mean and standard deviation) and non-interval variables (marital status, education, job, SES) were summarized as frequency counts and percentages. The non-interval variables included the age of beginning to smoke WT, the history of WTS, the type of tobacco, the history of WTS in other family members, the frequency of WTS per week. After examining the univariate regression results, the variables with a *p*-value lower than 0.2 in the univariate analysis were included in the multivariate analysis. The expected outcome was binary (0 for no cessation and 1 for cessation). To explain the predictive power of TPB constructs, at first, the model #1 was implemented in the presence of significant variables in multivariable logistic regression. Then, in model #2, the habit construct was removed and multivariable logistic regression was fitted. The data were statistically analyzed using STATA 14.2, and a *p*-value of < 0.05 was set as statistically significant.

## Results

Most women (46.5%) were between 31 and50-year-old age; 69.7% of the women were married and 50% had 1–15 years history of WTS smoking. Most women (70.4%) had smoked WTS 4–10 per week. According to the multivariable regression analytic results, the correlations between age, occupation, frequency of WTS per week and the constructs of habit, social norms, perceived control, attitude, knowledge, and intention to cease WTS knowledge with the cessation behavior were statistically significant. Thus, the odds of ceasing WTS were 3 times as high among workers as housewives. With every one unit of increase in the intention to cease WTS, the odds of cessation are increased for 29%. With an increase of one score in perceived behavioral control, the odds of cessation are increased for 17%. With an increase of one score in knowledge, the odds of cessation are increased for 4%. With an increase of one score in attitude, the odds of cessation are altered for 33%. With an increase of one score in the social norms, the odds of cessation are increased for 3%. Similarly, with any one unit of increase in habit, the odds of cessation are decreased for 36% (Table 2).

**Table 2:** Univariate and multivariate regression analysis of demographic variables and WTS cessation (*N* = 1764)

	**Variable**	**Number (%) total**	**Cessation N (%)**	**No cessation**	**Univariable**		**Adjusted**	
				**N(%)**	**OR (95% CI)**	***p* value**	**OR (95% CI)**	***p* value**
**Age**	15–30	561(31.8)	105(40.4)	456(30.3)	reference			
	31–50	820(46.5)	113(43.5)	707(47.0)	.69 (.52-.93)	0.014	0.12(0.05–0.28)	<0.001
	51–88	383(21.7)	42(16.2)	341(22.7)	.53 (.36—.79)	0.001	0.10 (0.03–0.31)	<0.001
**Marital status**	Single/divorced Widowed	535(30.3)	85(32.7)	450(29.9)	reference			
	Married	1229(69.7)	175(67.3)	1054(70.1)	.88(.66–1.16)	0.369		
**Educational level**	Illiterate	259(14.7)	34(13.1)	225(15.0)	reference			
	Primary school	395(22.4)	53(20.4)	342(22.7)	1.03(.65- 1.63)	0.915		
	Secondary school	436(24.7)	70(26.9)	366(24.3)	1.27(.81—1.97)	0.296		
	Diploma	438(24.8)	79(30.4)	359(23.9)	1.46(.94—2.25)	0.09		
	University	236(13.4)	24(9.2)	212(14.1)	.75(.43—1.30)	0.308		
**Occupation Status**	housewife	308(17.5)	56(21.5)	252(16.8)	reference			
	Working outside home	1456(82.5)	204(78.5)	1252(83.2)	1.36 (.98- 1.89)	0.061	3.70(1.89–7.24)	<0.001
**SES**	Very low	277(15.7)	40(15.4)	237(15.8)	reference			
	Low to medium	1294(73.4)	200(76.9)	1094(72.7)	1.08 (.75- 1.56)	0.67		
	High to Very high	193(10.9)	20(7.7)	173(11.5)	.68 (.39- 1.21)	0.194		
**Age beginning to smoke**	7–20	1002(56.8)	157(60.4)	845(56.2)	reference			
	21–40	735(41.7)	99(38.1)	636(42.3)	.84(.64- 1.10)	0.202		
	41–50	27(1.5)	4(1.5)	23(1.5)	.94(.32—2.74)	0.904		
**History of smoking**	1–15	882(50.0)	164(63.1)	718(47.7)	reference			
	16–30	663(37.6)	62(23.8)	601(40.0)	.45 (.33-.62)	0	0.79 (0.33–1.89)	0.601
	31–68	219(12.4)	34(13.1)	185(12.3)	.80 (.54—1.20)	0.291	1.60(0.59–4.31)	0.354
**Tobacco type**	Fruity	379(21.5)	46(17.7)	333(22.1)	reference			
	Local	1156(65.5)	176(67.7)	980(65.2)	1.30 (.92- 1.84)	0.139	1.25(0.55–2.84)	0.596
	Local-fruity	229(13.0)	38(14.6)	191(12.7)	1.44 (.90—2.29)	0.124	2.13 (0.86–5.28)	0.102
**Smoking by other family members**	NO	691(39.2)	95(36.5)	596(39.6)	reference			
	Yes	1073(60.8)	165(63.5)	908(60.4)	1.14 (.87- 1.50)	0.346		
**Smoking per week**	4–10	1242(70.4)	143(55.0)	1099(73.1)	reference			
	11 – 20	447(25.3)	70(26.9)	377(25.1)	1.43(1.05- 1.94)	0.024	1.56(0.92–2.62)	0.096
	21 – 70	75(4.3)	47(18.1)	28(1.9)	12.90 (7.83- 21.25)	0	3.33 (1.29–8.59)	0.013
	**Habit**	19.49 ± 4.97	12.63 ± 4.90	20.68 ± 3.91	.65(.62—.68)	0	0.63 (0.58–0.68)	<0.001
	**Social norms**	48.92 ± 18.65	66.36 ± 23.78	45.90 ± 15.77	1.06 (1.05- 1.07)	0	1.03 (1.01–1.05)	<0.001
	**Perceived control**	55.78 ± 13.34	66.72 ± 6.1	53.88 ± 13.34	1.39 (1.31- 1.49)	0	1.168(1.13–1.21)	<0.001
	**Knowledge**	14.16 ± 1.80	15.28 ± 2.38	13.97 ± 1.61	1.04 (0.97- 1.12)	0.227	1.05(1.01–1.09)	0.048
	**Intention**	8.50 ± 3.11	11.20 ± 4.10	8.04 ± 2.65	1.31 (1.26—1.36)	0	1.29(1.18–1.40)	<0.001
	**Attitude**	31.72 ± 8.29	39.53 ± 12.23	30.37 ± 6.51	1.12 (1.11- 1.14)	0	1.33 (1.16–1.54)	<0.001

The relationship between TPB constructs is reported in Table 3, and the results show a high enough correlation between the different constructs.

**Table 3:** Correlation and M SD of TPB contracts

	M ± SD	Cronbach’s alpha	1	2	3	4	5	6
**1.knowledge**	14.16 ± 1.81	0.419		.125^**^	-.177^**^	.109^**^	288^**^	.242^**^
**2.Perceived control**	55.78 ± 13.34	0.954	.125^**^		-.322^**^	.198^**^	.146^**^	.208^**^
**3.habit**	19.49 ± 4.97	0.886	-.177^**^	-.322^**^		-.243^**^	-.444^**^	-.458^**^
**4.Intention**	8.50 ± 3.12	0.96	.109^**^	.198^**^	-.243^**^		.319^**^	.277^**^
**5.attitude**	31.72 ± 8.30	0.863	.288^**^	.146^**^	-.444^**^	. .319^**^		.649^**^
**6.Social norm**	48.92 ± 18.65	0.977	.242^*^	.208^**^	-.458^**^	.277^**^	.649^**^	

** Correlation is significant at the 0.01 level (2-tailed)

* Correlation is significant at the 0.05 level (2-tailed)

Multivariate logistic regression was used to model the predictors of WTS. The independent variables included age, frequency of WTS per week, occupation, knowledge, attitude, control, perceived behavior, intention, habit and social behavior. In model #1, all the independent variables that were significant in the multivariate analysis were included in the model, and in model #2, the habit variable was excluded from the model (Table 4).

**Table 4:** Model comparison: Model #1 in the presence of habit and #2 without habit

model	Variable	OR (95% CI)	S.E	Z	*p*-value
`Model #1	Age				
	31–50	0.11 (0.06–0.22)	0.04	-6.43	<0.001
	51–88	0.10 (0.50–0.21)	0.04	-6.22	<0.001
	Weekly WTS				
	11–20	1.57 (0.94–2.64)	0.41	1.72	0.086
	21–70	3.23 (1.26–8.30)	1.56	2.43	0.015
	Occupation	3.00 (1.62–5.56)	0.94	3.49	<0.001
	Habit	0.63 (0.59–0.68)	0.02	-12.9	<0.001
	Social norm	1.03(1.01–1.05)	0.01	3.39	0.001
	Perceived control	1.17(1.13–1.21)	0.02	8.56	<0.001
	Knowledge	1.06(1.01–1.10)	0.022	2.64	0.008
	Intention	1.26 (1.17–1.37)	0.05	5.73	<0.001
	Attitude	1.31 (1.14–1.50)	0.9	3.93	<0.001
Model #2	Age				
	31–50	0.24(0.18–0.39)	0.06	-5.81	<0.001
	51–88	0.21(0.12–0.34)	0.05	-5.98	<0.001
	Weekly WTS				
	11–20	1.23(0.82–1.84)	0.25	0.99	0.322
	21–70	4.11 (1.94–8.72)	1.58	3.68	<0.001
	Occupation	2.20 (1.29–3.76)	0.6	2.88	0.004
	Social norm	1.04 (1.03–1.06)	0.01	6.29	<0.001
	Perceived control	1.14(1.11–1.17)	0.01	9.77	<0.001
	Knowledge	1.05(1.02–1.08)	0.02	3.02	0.003
	Intention	1.20(1.13–1.27)	0.03	5.96	<0.001
	Attitude	1.30 (1.17–1.45)	0.07	4.83	<0.001

The fit indices of each model were calculated and based on the fit indices, model # 1 was chosen as the best final model (Table 5).

**Table 5:** Comparative Fit indices: Model # 1 in the presence of habit and model # 2 without habit

Model	Pseudo R^2^	AIC	BIC	Accuracy	Specificity	Sensitivity	Chi2 (*p*-value) Hosmer-lemsh
Model #2	0.447	837.8865	898.1152	90.70%	97.67%	50.38%	95.62 *p* < 0.001
Model #1	0.6577	528.9365	594.6405	95.69%	98.87%	77.31%	85.44 *p* < 0.001

**Fig. 3:**
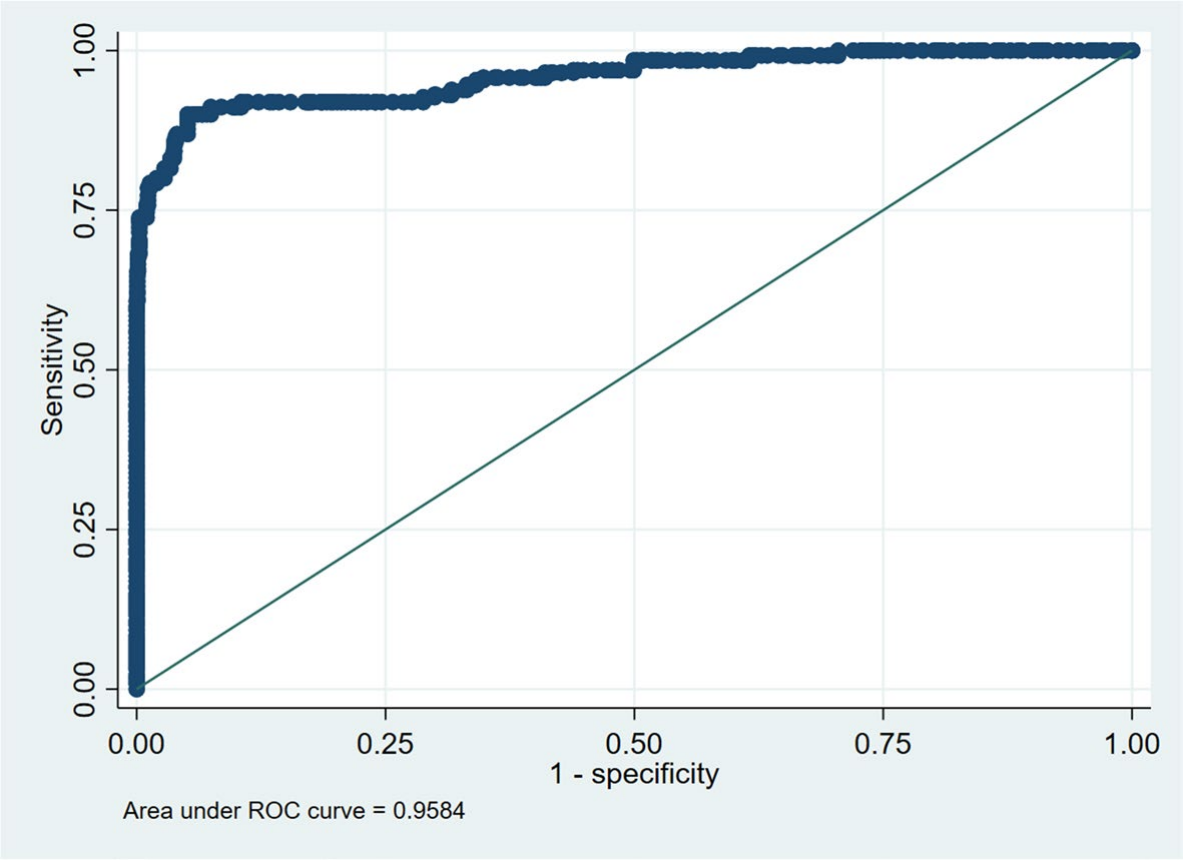
ROC curve plot for model #1

**Fig. 4:**
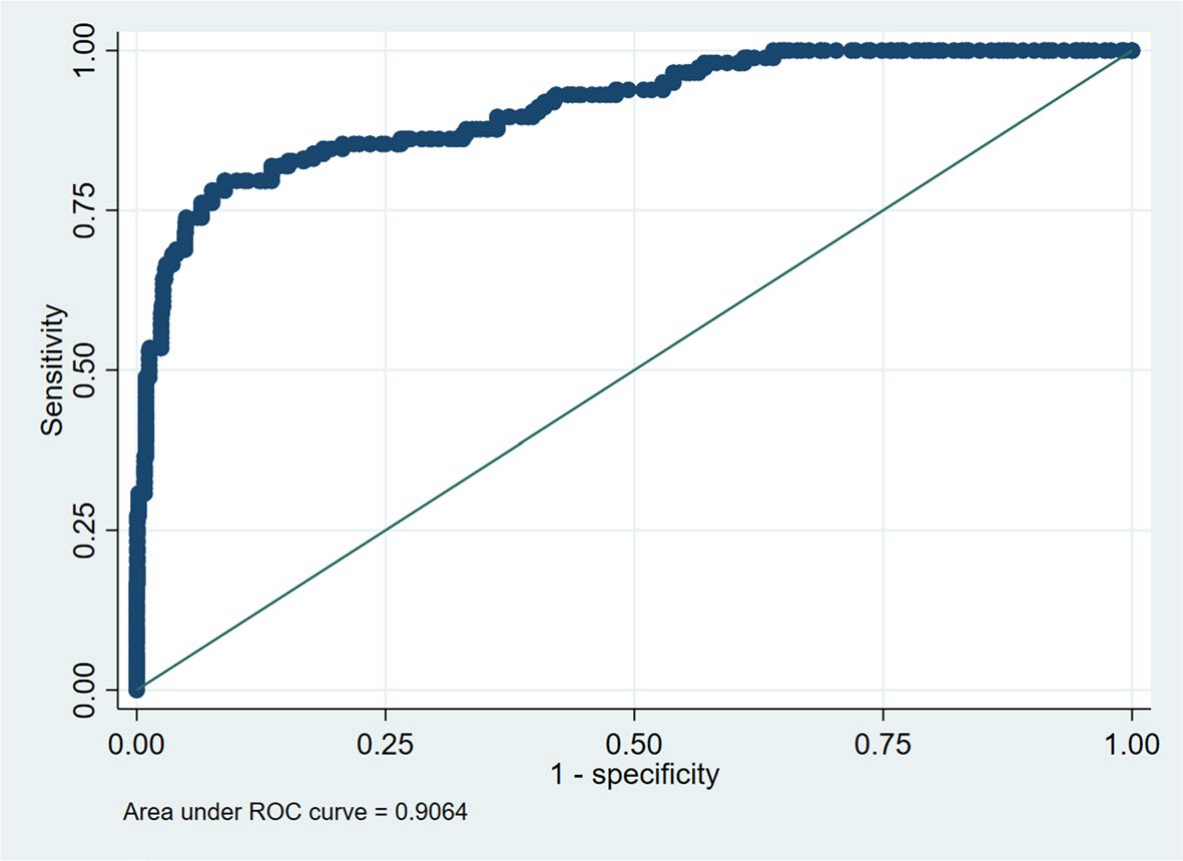
ROC curve plot for model #2

The ROC curve in Fig. 3, and Fig. 4 show that model #1 had a better fit than model #2.

## Discussion

The present research is pioneering in predicting waterpipe cessation behavior in two groups of women who successfully quit waterpipe and women who used waterpipe based on the TPB theory plus the additional construct of habit.

In the present study, the mean scores of all constructs in women who ceased WTS were higher than those who smoked WT. Attitude, intention, knowledge, perceived behavior control, social norms and habit were the best predictors of WT cessation, respectively. Also, among the demographic variables, occupation, weekly WTS and age were, respectively, the main predictors of WT cessation.

As the present findings showed, the attitude was the foremost predictor of WT cessation in women. This finding indicates that women had a positive appraisal of WT cessation and were aware of the benefits of cessation. One assumption of the TPB is that before deciding to adopt a behavior, people first check its application and efficiency and if they perceive the behavior reasonable, they adopt it. According to Ajzen, a change in attitude leads to a change in intention, which affects the behavior [47]. Similar to the present findings, in other studies, attitude was among the predictors of smoking cessation [29, 48]. Therefore, any attempt to change women’s attitude towards the high risk of WT can be helpful.

Next to the attitude, intention was the strongest predictor of WT cessation in women. As the analysis showed, it can be concluded that the negative attitude towards WTS has increased the intention of WT cessation in women. Consistent with these findings, WTS behavior was predicted by intention [28, 49]. In this regard, Ajzen and Fish reckoned that intention predicts actual behavior [50]. Moreover, the intention is the pre-action stage and does not always lead to the behavior in someone ready to adopt the behavior, because internal and external factors over time can change one’s intention to show behavior. Therefore, it is necessary to consider all the above-mentioned factors to change intention into an actual behavior.

As the findings revealed, the knowledge construct ranked third among the predictors of WTS. It can be argued that increasing knowledge of the adverse effects of WTS or the benefits of cessation can be the basis of WT cessation. Moreover, it can be argued that knowledge can play an important role in forming attitudes and behaviors. Adoption of new behaviors will be easier if it is based on correct knowledge or awareness and positive attitudes. Consistent with the present findings, a qualitative study found that awareness of the potential harms of WT was a factor contributing to WT cessation [51]. In another study, the increase in WTS in women is due to a lacking knowledge of its adverse effects [44]. Also, another study showed that pregnant women with second and subsequent pregnancies showed a greater willingness to cease WT than the virgin due to their experience and knowledge of the adverse effects [52]. Therefore, raising women’s knowledge of the adverse effects of WTS and the benefits of cessation should be a priority for local health policy makers.

Next to knowledge was the PBC as the strongest predictor of smoking cessation in women. Consistent with the present findings, perceived behavioral control could predict WT cessation [29, 49, 53]. In some research, Zhao et al. considered the role of perceived behavioral control in smoking behavior and intention to be very strong [49].

As pinpointed in the TPB, human behavior is led by beliefs about the underlying existence of variables that can facilitate or impede the emergence of particular behaviors. These beliefs might contribute to PBC, which not only directly anticipates intentions but also anticipates the behavior. Those with a high level of PBC seem to be better motivated to show particular behaviors and show the actual behavior. Such influences have physiological roots. Particularly, the low PBC has shown to lower the activity of brain which is correlated with the motor development and emergence of a given behavior [54, 55].

This finding points to the fact that PBC can be an important and influential variable in WT cessation. Therefore, in designing effective interventions to cease WTS, special attention should be paid to this variable.

Next to the perceived behavioral control was social norms to predict the WT cessation behavior in women. It can probably be claimed that an important factor that can encourage a smoker to cease WTS is the motivating role of important others in her life. This is probably the cheapest way to help people cease WT. Some research showed that those highly motivated to cease smoking are 4 times as successful in cessation as the less motivated [56]. Similarly, in another study, the advice of important others was reported as an important factor affecting the intention to cease smoking [57]. Yet, in some other studies, social norms did not predict the intention to cease smoking [28, 29, 49]. In Zhao's study, subjective norms construct was found to be the most difficult construct to change in the intervention [58]. These divergent findings can be partially attributed to the different socio-demographic characteristics of the research population, the study design and the type of tobacco substance. Therefore, using this strategy means addressing important and influential people in women's lives as their persuasive and motivating role can have a significant effect on WT cessation.

The present findings showed that habit is a predictor of WTS in women. The habit of WTS shows a kind of physical and psychological dependence on smoking and seems to be a major barrier to WT cessation. Consistent with these findings, in another study, nicotine dependence was considered a major barrier to strengthening PBC compared to smoking cessation. Therefore, although nicotine-dependent smokers make more efforts to cease smoking than non-dependent smokers, they are less successful in cessation [59]. Other studies reported that higher levels of nicotine dependence are associated with the intention to cease and also with less self-efficacy [60, 61]. Gupta's study showed those who do not cultivate the habit of WTS can be successful against smoking [62]. Of note is that the habit construct increased sensitivity for 27% and accuracy for 5% in the model. This finding shows in future interventions to cease smoking, special attention should be paid to the effective factors involved in habit formation, their adjustment and the design of specific interventions. Therefore, great attention should be paid to the habit construct in predicting the intention and behavior to take an effective step in improving the health behavior as much as possible by implementing interventions to stop the habits in the past.

According to the present findings, the odds of WT cessation among working women were three times as high as housewives. Consistent with these findings, in a qualitative study, women's occupation was a factor that encouraged the participants to cease smoking and reduce the use of WTS [51]. A study showed that unemployment reduces the chances of smoking cessation, and conversely, having fun in a professional job can increase the chances of cessation [63]. Arguably, someone who is unemployed is likely to have more free time. As a result, she is more likely to smoke WT in her free time. Also, working women are more pressed for time, and as a result tend less to smoke WT while doing their job at certain hours of the day and also having limited free time.

According to the present findings, the older women had lower odds of WT cessation. It can be argued that probably the WTS behavior has been institutionalized over many years and has led to physical and psychological dependence in women, and as a result, has made the act of cessation difficult. Thus, the chances of cessation or any attempts to cease WT are decreased [64].

According to the present findings, the odds of WT cessation are higher in women with a high frequency of WTS than those with a low frequency of WTS. Concerning the more consumption and the higher odds of cessation, it can be concluded that the women have probably experienced the physical effects of the frequent smoking of WT. Thus, they have faced the physical consequences of waterpipe, and this has motivated them to cease WT smoking. In line with these findings, in another study, health concerns were the most important reason for ceasing smoking. Approximately 64.3% of smokers with chronic diseases tried to quit smoking, and approximately 21.0% quit smoking successfully [65].

### Implications

The present findings can be a guide for researchers to design effective and goal-oriented interventions to cease smoking at the lowest cost and as fast as possible. The present findings can be a basis for comparison with the results of future research in this field. Also, the findings can help policy makers and health doctors implement behavioral interventions based on the theory and, thus, help to come up with the best possible effective solutions to WT cessation.

### Strengths and limitations of the study

There were several limitations in the present study. First, as the study was a cross-sectional survey, it precluded any meaningful discussion about causality.

The questions were completed as self-reports. Thus, it was possible that the women give socially favorable answers. The researcher tried to minimize this bias by ensuring the confidentiality of the information. In this research, the male population was not included, so the generalizability of findings is limited. However, these results can be generalized to Middle Eastern women who are similar in terms of culture and the prevalence of WTS. Failure to do biochemical tests to confirm smoking cessation was one limitation of the current research, although from the beginning, it was attempted to reduce the effect of this bias by increasing the trust and intimacy between the researcher and the women. Despite the limitations, the current research had several strengths. It was pioneering in predicting the actual behavior of WTS in women using the TPB constructs. This research and its findings can pave the way for the future line of inquiry. Adding the habit construct, which can be an important variable in predicting smoking cessation, was another strength of the present study. This study was conducted in an area where WTS was highly prevalent. Women who had a long history of WTS were included in the study. Also, the women who successfully ceased smoking were those whose cessation had lasted for at least 6 months.

## Conclusions

The current research proved the effectiveness of the TPB model in predicting waterpipe cessation behavior. The knowledge provided by this research can help develop a systematic and effective intervention to facilitate waterpipe cessation. Focusing on the habit variable can have a very effective role in quitting waterpipe smoking in women, so that the inclusion of this variable in the model improved the fit of the model by 21%. The use of the current model can be a useful tool for health professionals as a theoretical framework in the prevention and reduction of waterpipe use.

## Data Availability

The datasets generated during and/or analyzed during the current study are available from the corresponding author on reasonable request.

## Abbreviations

PBC: Perceived behavior control
TPB: Theory of Planned Behavior
WT: Waterpipe
